# A novel conserved family of Macro-like domains—putative new players in ADP-ribosylation signaling

**DOI:** 10.7717/peerj.6863

**Published:** 2019-05-01

**Authors:** Małgorzata Dudkiewicz, Krzysztof Pawłowski

**Affiliations:** 1 Department of Experimental Design and Bioinformatics, Faculty of Agriculture and Biology, Warsaw University of Life Sciences, Warszawa, Poland; 2 Department of Translational Mecicine, Clinical Sciences, Lund University, Lund, Sweden

**Keywords:** Bioinformatics, Protein structure prediction, Uncharacterized proteins, Novel enzyme families, Intellectual disability, Macrodomain ADP-ribosilation, DUF2362

## Abstract

The presence of many completely uncharacterized proteins, even in well-studied organisms such as humans, seriously hampers a full understanding of the functioning of living cells. One such example is the human protein C12ORF4, which belongs to the DUF2362 family, present in many eukaryotic lineages and conserved in metazoans. The only functional information available on C12ORF4 (Chromosome 12 Open Reading Frame 4) is its involvement in mast cell degranulation and its being a genetic cause of autosomal intellectual disability.

Bioinformatics analysis of the DUF2362 family provides strong evidence that it is a novel member of the Macro clan/superfamily. Sequence similarity analysis versus other representatives of the Macro superfamily of ADP-ribose-binding proteins and mapping sequence conservation on predicted three-dimensional structure provides hypotheses regarding the molecular function for members of the DUF2362 family. For example, the available functional data suggest a possible role for C12ORF4 in ADP-ribosylation signaling in asthma and related inflammatory diseases.

This novel family appears to be a likely novel ADP-ribosylation “reader” and “eraser,” a previously unnoticed putative new player in cell signaling by this emerging post-translational modification.

## Introduction

In 2019, there are still 3918 protein families and three protein clans in the Pfam database (http://pfam.xfam.org) that are described simply as “DUFs”–domains of unknown function. These contain together over 1.3 million amino acid sequences, and as many as 512 (13%) Pfam “DUF” families contain human protein sequences. Proteins containing such domains are usually disregarded by biologists (Pawlowski, 2008) although often they turn out to be important signaling enzymes (e.g. Dudkiewicz et al., 2012; Sreelatha et al., 2018).

Recent findings of two independent groups from Saudi Arabia and Europe analyzing multiplex consanguineous families with members suffering from intellectual disability (ID) using whole-exome sequencing implicate the mysterious C12ORF4 as a causative gene for autosomal ID, an important health problem in society (Alazami et al., 2015; Philips et al., 2017). Genetic causes of ID still remain poorly explained because of its vast heterogeneity (Anazi et al., 2017). The involvement of C12ORF4 in ID was confirmed by observation of the presence of specific mutations in this protein-coding gene in all ID affected members of the analyzed families, including a missense variant (L328P), a deletion (Q267fs) and a frameshift. Another study shows that, in rats, the gene orthologous to C12ORF4 encodes a cytoplasmic protein, involved in mast cell degranulation (Mazuc et al., 2014).

In this study, we present evidence that C12ORF4 is likely involved in ADP-ribosylation of proteins, an important post-translational modification that plays a key role in signal transduction in many important cellular processes such as DNA repair, regulation of transcription, maintenance of chromatin stability, cell differentiation and proliferation, necrosis and apoptosis (Han, Li & Fu, 2011; Palazzo, Mikoč & Ahel, 2017; Cohen & Chang, 2018; Lüscher et al., 2018). NAD+ is an essential cofactor and donor of the ADP-ribose group in a reaction which starts with breaking the bond between nicotinamide and ribose. As a result of the ADP-ribosylation reaction, nicotinamide and ADP-ribosylated proteins are released. Errors in the ADP-ribosylation of proteins can be associated with cancerogenesis processes (Barkauskaite et al., 2013). ADP-ribosylation of DNA is relatively less common and until recently has only been described for a small number of bacterial toxins (Jankevicius et al., 2016). However, there are new reports showing DNA ADP-ribosyltransferase activities of some mammalian PARPs (Munnur & Ahel, 2017; Zarkovic et al., 2018).

One of the important players in ADP-ribosylation, the Macro domain, is defined as a module of about 180 amino acids which can bind the important NAD metabolite–ADP-ribose–or related ligands (Rack, Perina & Ahel, 2016; Rosenthal et al., 2013). The Macro domain was originally named A1pp, as it was described in association with ADP-ribose 1-phosphate processing activity in yeast (Martzen et al., 1999), but later it was renamed Macro in reference to the fact that it makes up the C-terminal domain of mammalian core histone macro-H2A (Allen et al., 2003; Karras et al., 2005). Macro domain proteins are widespread in eukaryotes, but also present in pathogenic bacteria, archaea and in ssRNA viruses, such as coronaviruses, Rubella and Hepatitis E viruses (Feijs et al., 2013; Rack, Perina & Ahel, 2016).

The Macro domain is a universal “tool” for recognition of the ADP-ribose nucleotide and its polymers found in a number of otherwise unrelated proteins, which belongs to a cellular toolkit used for ADP-ribosylation of the proteome and its reversion. The typical three-dimensional structure of the Macro domain can be described as a mixed alpha/beta/alpha fold. A six-strand beta sheet is sandwiched between five α-helices with the ligand binding pocket lying between two loops (loop 1 and loop 2) (Slade et al., 2011; Rack, Perina & Ahel, 2016). Some Macro domains are shorter and lack either the first strand or the C-terminal helix 5. Well-conserved residues build a hydrophobic cleft and cluster around the ADP-ribose binding site. Most important for ligand binding by Macro domains are conserved aromatic residues in binding pocket (π–π stacking interactions with the adenosine ring) and the aspartate residue coordinating the N6 atom of adenosine. The pyrophosphate and distal ribose are stabilized by two binding loops, the first of which can contain catalytic residues in cases when the Macro domain exhibits hydrolase activity.

Phylogenetic analysis of the Macro clan/superfamily distinguishes six classes of Macro domains: MacroD-type and MacroH2A-like (Macro family in the Pfam database, PF01661), Macro2-type (Macro_2, PF14519), ALC1-like, PARG-like (PARG_cat, PF05028) and SUD-M like (SUD-M, PF11633) (Rack, Perina & Ahel, 2016). The Pfam database additionally lists the M17 aminopeptidase N-terminal domain (PF18295 and PF02789) and DUF2362–an uncharacterized domain conserved in bacteria (PF10021)–as members of the Macro clan.

Depending on their functions, Macro domains can be divided into two distinct groups–“readers” and “erasers”–where the latter completes the “writer-reader-eraser” ADP-ribosylation triad, whereas the “writers” are ADP-ribosyltransferases. In humans, both “readers” and “erasers” are found in the Macro family (PF01661) (Chen et al., 2011; Barkauskaite et al., 2013; Jankevicius et al., 2013), while eraser-type PARG-like domains belong to a separate PARG-cat family (PF05028, poly-ADP-ribosyl-hydrolases) (Slade et al., 2011; Dunstan et al., 2012). Reader domains are found in PARPs (multidomain enzymes which catalyze mono-and poly-ADP-ribosylation of proteins) (Gibson & Kraus, 2012; Langelier et al., 2018), in histones MacroH2A (1 and 2) (Posavec, Timinszky & Buschbeck, 2013), in ALC1 (Lehmann et al., 2017) and GDAP2 (Neuvonen & Ahola, 2009) proteins and have no catalytic function, they accompany other domains and serve for recognition of ADP-ribose moieties.

Erasers have ADP-ribosyl-hydrolase activity and are found in mono-(MacroD1/D2, TARG) and poly-ADP-ribosyl-hydrolases (PARG proteins).

The mechanisms of ligand binding in both readers and erasers are similar, although reader Macro domains coordinate ADPr in a rather relaxed conformation while some erasers (e.g., MacroD2) keep ligands in strained form due to the presence of a structural water molecule in the binding cleft. However, the main difference between these two classes lies in the presence of catalytic residues in loops 1 or 2 of the “erasers.” The structure and reaction mechanisms of the human hydrolytic Macro domains represent three different types.

**Figure 1:**
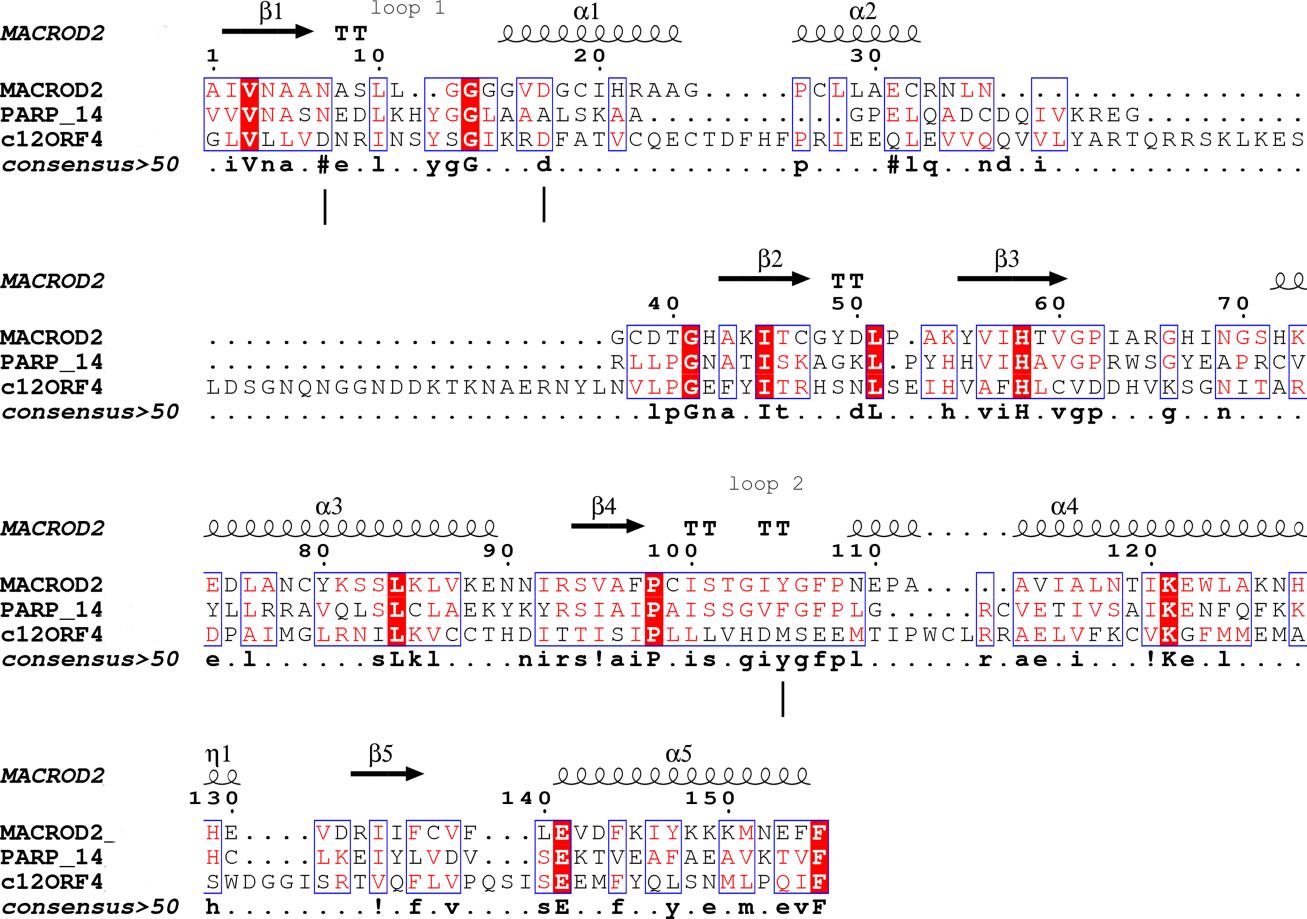
Sequence alignment for human amino acid sequences of C12ORF4, PARP14 and MacroD. Alignment constructed using Phyre^2^ and HHpred algorithms. Secondary structure elements derived from human MacroD1 PDB structure are shown above, vertical lines indicate positions of catalytic and ligand binding residues Asn92, Asp102 and Tyr190 in human MacroD2 sequence and their possible counterparts in C12ORF4.

Type-1: MacroD1/2 (Chen et al., 2011; Barkauskaite et al., 2013; Jankevicius et al., 2013): (*O*-acetyl-ADP-ribose deacetylase)-ADPr is bound in a constrained conformation forced by the presence of a structural water molecule and highly conserved aromatic residues located in MacroD-type signature motifs Nx(6)GG[V/L/I]D and G[V/I/A][Y/F]G identified in loops 1 and 2 (Fig. 1), respectively. Catalytic residues for hydrolysis reaction, Asn and Asp, are anchored in loop 1 and interact electrostatically with the distal ribose.

Type-2: TARG1 (Sharifi et al., 2013): (Terminal ADP-ribose protein Glycohydrolase 1)-ADPr binding cleft more closely resembles the “reader” ligand binding site and stabilizes the substrate in a relaxed conformation. Catalytic residues are lysine and aspartate, located in loops 1 and 2, respectively.

Type-3: PARG (Slade et al., 2011; Dunstan et al., 2012): (Poly(ADP-Ribosyl) Glycohydrolase) has a three-domain structure: a Macro domain together with a TARG accessory domain form the canonical catalytic core, the third domain is a putative regulatory region. The PARG Macro domain contains a specific catalytic motif within loop 1, and the main catalytic residues are glutamate in loop 1 and phenylalanine in loop 2. PARG is predominantly an exoglycohydrolase with only minor contributions of endocleavage activity.

Macro domains are a very divergent and evolutionarily old protein fold, their common feature is affinity to ADPr. The same function can be realized using different mechanisms and binding modes, but overall the Macro domain fold still remains recognizable.

In this paper, we present Macro domain-like structural predictions for the uncharacterized C12ORF4/DUF2362 family. We also discuss the relevance of the structural predictions for the likely molecular function in the DUF2362 family. Furthermore, we analyze the phylogenetic spread of the C12ORF4 genes and their relationship to members of the Macro superfamily/clan. We also summarize and analyze structural features of possible ligand binding site and catalytic loop in the newly identified Macro domain using models constructed for its human C12ORF4 representative. Nevertheless, we stress that this in silico prediction report should be the starting point for experimental validation of the structural and functional hypotheses.

## Materials and Methods

Initial analysis of distant sequence similarities of human C12ORF4 and the DUF2362 family was performed using three state-of-the-art servers for protein structure and function prediction: Phyre2 (Protein Homology/analogY Recognition Engine V 2.0) (Kelley et al., 2015), FFAS03 (Fold and Function Assignment System) (Jaroszewski et al., 2011), and HHpred (Homology detection & structure Prediction by HMM–HMM comparison) (Soding, Biegert & Lupas, 2005), that implement Hidden Markov Models, profile–profile alignment methods, and related approaches for the detection of remote sequence similarity.

To assess phylogenetic spread of the DUF2362 family, the Pfam Representative Proteome rp75 sequence collection was chosen (Chen et al., 2011). Sequences were next classified and described using UniProt database Taxonomy tools, together with GeneTree (Vilella et al., 2009) and TreeFam (Ruan et al., 2008) gene orientated phylogenetic databases, providing curated phylogenetic trees for animal gene families. Thus, 381 sequences from 261 species were assigned to taxonomic groups and visualized using the iToL server on a dendrogram representing the Tree of Life (Letunic & Bork, 2016).

To assess similarities in the amino acid sequences of human C12ORF4 and PARP14/MacroD2 sequences, a multiple sequence alignment was constructed using the HHpred and Phyre2 algorithms, and manually adjusted. Secondary structure assignment from the PDB structure of MacroD2 (4IQY) was added to the final alignment. To graphically represent the resulting alignment, ESPript 3.0 software was used (Robert & Gouet, 2014).

A sequence logo for the rp75 set of DUF2362 sequences was created using the Pfam alignment. The alignment was edited to adjust it to the selected “master sequence” by removing columns containing gaps in human C12ORF4. Graphical representations of the sequence logo were prepared using the WebLogo3 server (Schneider & Stephens, 1990).

The structures of human MacroD2 (PDB code: 4IQY) and PARP14 (PDB code: 3Q6Z) were used to depict similarities between these proteins and human C12ORF4 according to BLOSUM62 matrix values (from 0 to 11) in pairwise alignments with human C12ORF4. High positive BLOSUM62 values signify residues which are identical or highly similar and frequently conserved by evolution.

In order to visualize sequence variation in the DUF2362 family, conservation of sequence positions was mapped onto known Macro domain structures as well as on the structure model of human C12ORF4. Conservation values derived from the Jalview alignment editor (Waterhouse et al., 2009) were automatically calculated as quantitative alignment annotation measuring the number of conserved physicochemical properties for each column of the alignment (Livingstone & Barton, 1993).

A protein three-dimensional structure model for human C12ORF4 was built using the homology modeling method in Modeller9v21 (Webb & Sali, 2014) based on a human MacroD2 structure template (PDB ID: 4IQY). The loop (374–408) that had no counterpart in the template was refined using the I-Tasser (Iterative Threading ASSEmbly Refinement) server for protein structure and function prediction (Yang et al., 2015). The putative ligand binding site in the modeled structure was identified using the COFACTOR algorithm (Zhang, Freddolino & Zhang, 2017), which, based on threading of the query structural model through the BioLiP protein function database, annotates proteins and identifies potential functional sites by local and global structure matches. Structures were visualized and analyzed using UCSF Chimera tools (Pettersen et al., 2004).

For survey of similarities between the Macro domain clan and C12ORF4 homologs, the CLANS algorithm (Frickey & Lupas, 2004) was run. The set of sequences selected for analysis included all the Pfam rp15 sequences from the six families of the Macro clan (CL0223)–Macro (PF10021), Macro2 (PF14519), PARG-cat (PF05028), PeptidaseM17_N, (PF02789) SUD-M (PF11633), DUF2263 a.k.a. bacterial-type PARG (PF10021) (Slade et al., 2011)–and the novel DUF2362 family (PF10154). ALC1-like sequences were added manually, because they are not represented in Pfam. An ALC1 homolog collection was created based on BLAST search results for the human CHD1L (ALC1) Macro domain. Resulting sequences were clustered using CDHIT with sequence identity cutoff 0.9 and thus 3,432 representative sequences were obtained. For the CLANS run, relationships from a single iteration of BLAST were used, up to the *E*-value of 1, thus including also sub-significant similarities. The BLOSUM62 substitution matrix was used in BLAST. To find more potential members of the DUF2362 family, the Jackhmmer algorithm was applied (Finn et al., 2015). The interactors of C12ORF4 were elucidated using the STRING database (Szklarczyk et al., 2015) and the Ingenuity system (Qiagen, Hilden, Germany).

## Results and discussion

### Assignment of DUF2362 proteins to the Macro clan

Analysis of distant similarities between human proteins and ADP-ribosylation-related families performed using the FFAS server suggested that the uncharacterized family DUF2362 might be similar to Macro domains. Table 1 shows that both FFAS and two other popular structure prediction servers predicted a weak but suggestive similarity to Macro for the human C12ORF4 protein (see also Fig. 1). These methods use sequence profiles or Hidden Markov Models to detect distant sequence similarities compatible with similar structures. Phyre^2^ and HHpred are remarkably concurring in their prediction of structural similarities for C12ORF4, identifying as best-scoring structure modeling templates the Macro domains of the human poly(ADP-ribose) polymerase 14 (PDB: 3Q6Z) and human protein-proximal ADP-ribosyl-hydrolase MacroD2 (PDB: 4IQY) or human MacroD1 (PDB: 2X47). Using the servers’ results, it was difficult to distinguish between the predictions, as confidence values were very close. However, FFAS03 ran on DUF2362 multiple sequence alignment used as the query gave the highest score for human PARP14 (PDB: 3Q6Z) as structural prediction, but did not pick human MacroD1/2 structures as significant hits. Using HHpred and Phyre^2^ local HMM profile–profile alignment, an alignment for human C12ORF4 and the two best hits was constructed, considering secondary structure assignments from the human MacroD2 structure (Fig. 1). Manual adjustment was performed only in the loop 1 region. Investigating similarities between C12ORF4 and important regions of MacroD2, a similarity was noticed to the characteristic MacroD motif Nx(6)GG[V/L/I]D in the C12ORF4 region aligned with loop 1. However, there is a difference; whereas the first residue in the glycine dyad is substituted by serine: Nx(6)SG[V/L/I]D. MacroD2 residues N92 (matched with D333 in human C12ORF4) and D102 (aligned to D345 in human C12ORF4) are catalytic amino acids electrostatically interacting with the distal ribose ring of ADPr ligands. Conservation of these residues in C12ORF4 seems to support its similarity to the catalytic Macro domain class, albeit tyrosine 190, crucial for eraser function of MacroD, is substituted by a methionine (M478). The structural water molecule and aromatic ring of Y190 located in eraser loop 2 bind the ADPr moiety in constrained position enabling initiation of its hydrolysis. The reader-type Macro domain in PARP14 has conserved Asp residues in loop 1, but tyrosine within the G[IV]YG motif is replaced by a phenylalanine, also possessing an aromatic ring. In C12ORF4, there is a matched methionine at this position. This brings uncertainty regarding the catalytic activity of the C12ORF4 protein, taking into account the fact that Y->F substitution in this motif can convert a catalytically active Macro domain to a reader-only pseudoenzyme (Rack, Perina & Ahel, 2016).

**Table 1:** Structure predictions for human C12ORF4 protein.

Template PDB id	Alignment coverage (% of C12ORF4 sequence)	Phyre2 confidence	FFAS03 score	HHpred confidence	Sequence identity %	HHpred *E*-value	Template info
3q6zA	39% (327–540)	98.1	−11.8	96.6	15	0.0088	Poly(ADP-ribose) polymerase 14
4iqyB	39% (327–540)	97.8	–	97.14	16	0.0011	*O*-acetyl-ADP-ribose deacetylase macrod2
2x47A	24% (410–540)	97.7	–	96.64	14	0.0047	Macro domain-containing protein 1

**Note:**

Phyre2, FFAS03 and HHpred results for human C12ORF4. Scores of the best PDB hits provided. Prediction confidence as %.

### Phylogenetic spread of the DUF2362 family and relationship to the Macro clan

**Figure 2:**
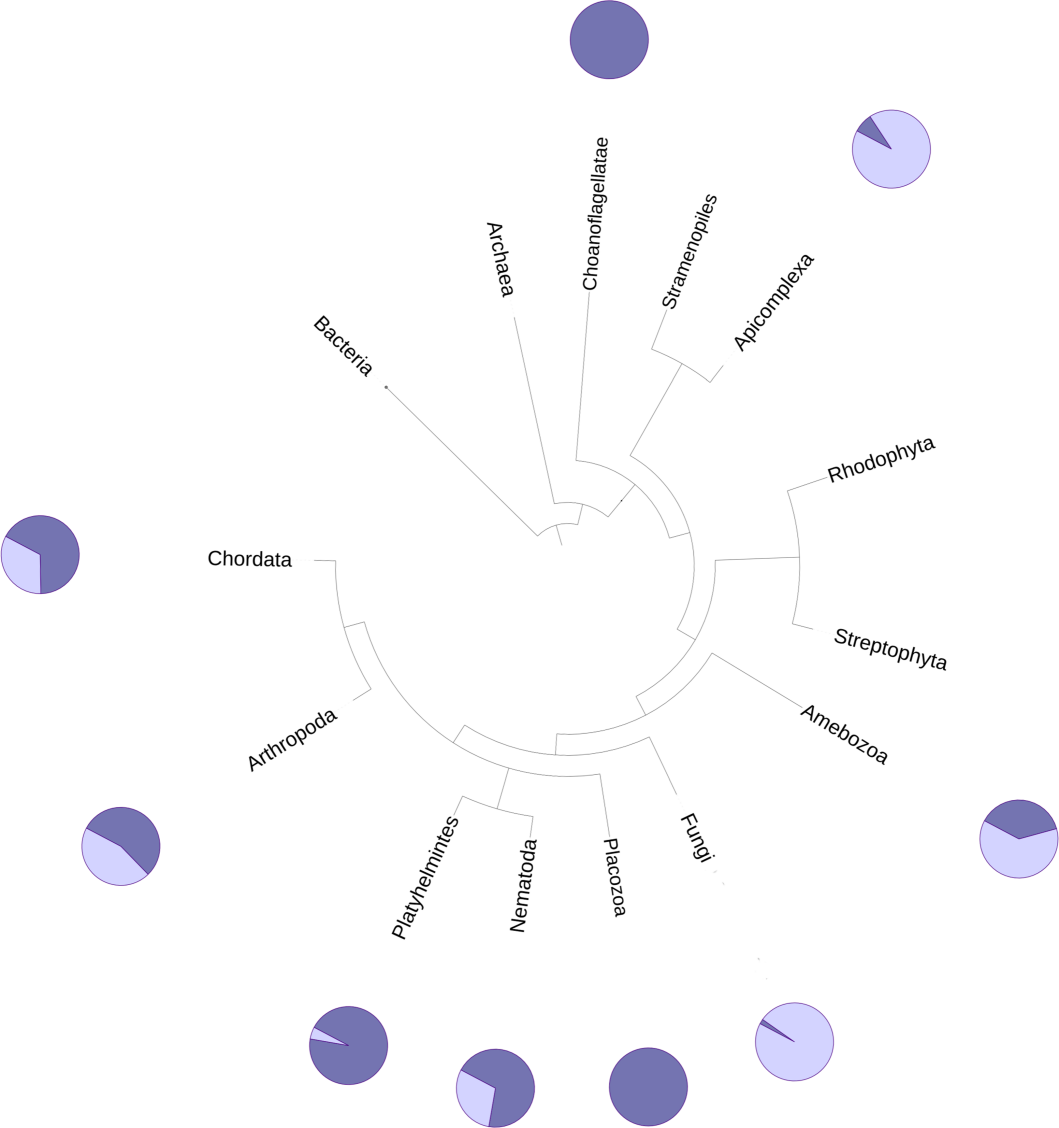
Phylogenetic spread of DUF2362 family. Selected branches of the universal Tree of Life. The dark blue sectors within the circles represent the percentage of representative genomes in the given taxonomic group possessing C12ORF4 homologs, as identified by Jackhmmer.

The DUF2362 family in the Pfam database contains sequences belonging to 261 species (see Fig. 2), mainly Metazoa, including the early Metazoan *Trichoplax adherens* (Placozoa), and also the closest unicellular relative of metazoans, *Capsaspora owczarzaki* (Ichthyosporea). DUF2362 is also found in some fungi (albeit the fungal C12ORF4 homologs belong to specialized atypical groups of fungi, e.g., Mucoromycota, Chytridiomycota, Zoopagomycota and Cryptomycota), Amoebozoa, Apicomplexa, Choanoflagellida and Cryptophyta. C12ORF4 homologs have not been found in most fungi, or in plants, archaea or bacteria. Overall, the Jackhmmer search for new potential family members also picked up 410 C12ORF4 homologs in the Representative Proteomes and about 700 C12ORF4 homologs in UniProt. The broad phylogenetic spread of this protein family confirms its ancient origin and potentially important cellular function. It is suggestive of a DUF2362 member being present in the last eukaryotic common ancestor and subsequent DUF2362 gene losses in some major eukaryotic lineages.

**Figure 3:**
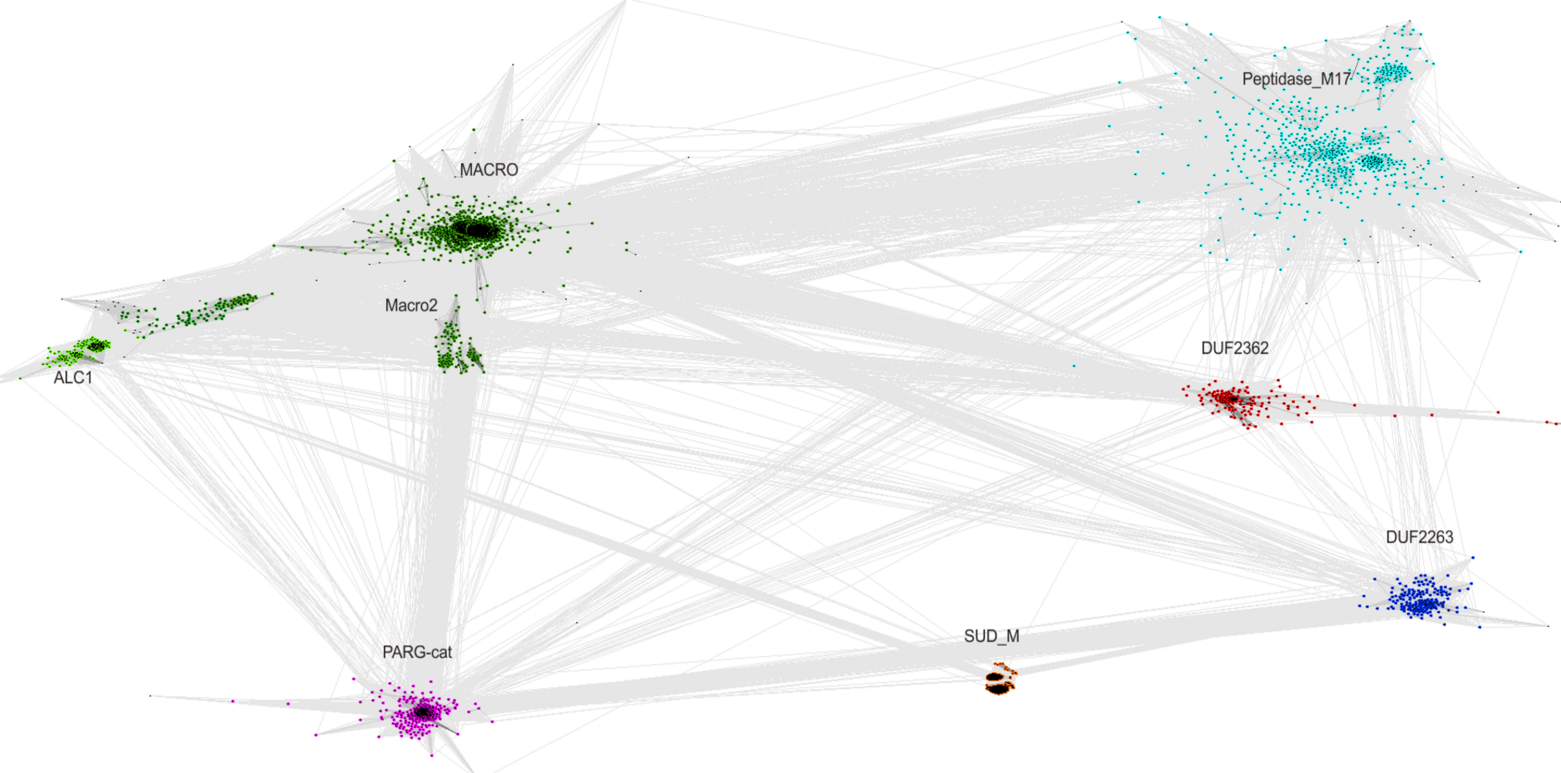
CLANS graph visualizing BLAST-detected sequence similarities among proteins of the Macro domain superfamily. CLANS clustering results for 3,232 representatives of the Macro domain superfamily (Pfam clan CL0223 plus ALC1 homologs and the DUF2362 family).

The relationship of the DUF2362 family to the Macro clan can be visualized using a graph-based approach: the CLANS algorithm (Frickey & Lupas, 2004). The CLANS graph depicts proteins as nodes connected by edges representing BLAST-detected significant or sub-significant sequence similarities. The nodes are clustered by “attractive forces” representing BLAST *E*-values (see Fig. 3). DUF2362 appears to be connected not only to the main family of the clan–Macro (PF01661), but also Macro2, Peptidase_M17, PARG-cat and DUF2263. The biggest grouping of the superfamily, the Macro family, contains the majority of known human Macro domains, eraser and reader-like, except one that belongs to the PARG family, poly(ADP-ribose)-glycohydrolases.

### Sequence variability in the DUF2362 family

While distant sequence similarity search tools strongly suggested the presence of a Macro-like domain in the C-terminal region of C12ORF4 (approx. residues 322–552), the presence of another domain in N-terminal part of the protein could not be established with confidence. HHpred reported a non-significant similarity to human all-helical nuclear pore complex protein Nup155, while FFAS03 reported a weak similarity to bacterial colicin E9.

**Figure 4:**
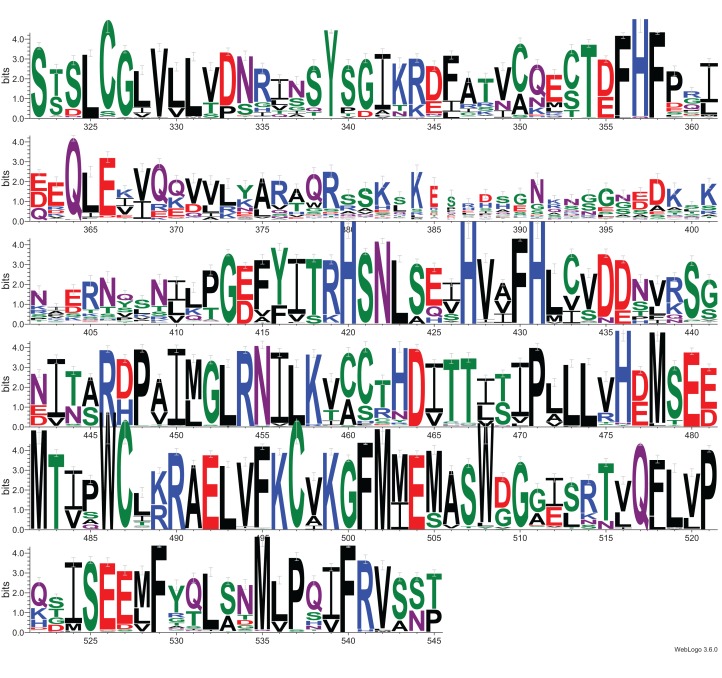
Sequence logo for Macro-like domain of the DUF2362 protein family. Multiple sequence alignment from the Pfam database (representative proteomes rp75 set), the logo was created for regions corresponding to positions 323–545 in human C12ORF4 amino acid sequences with Macro domain signal identified using remote protein homology detection tools.

**Figure 5:**
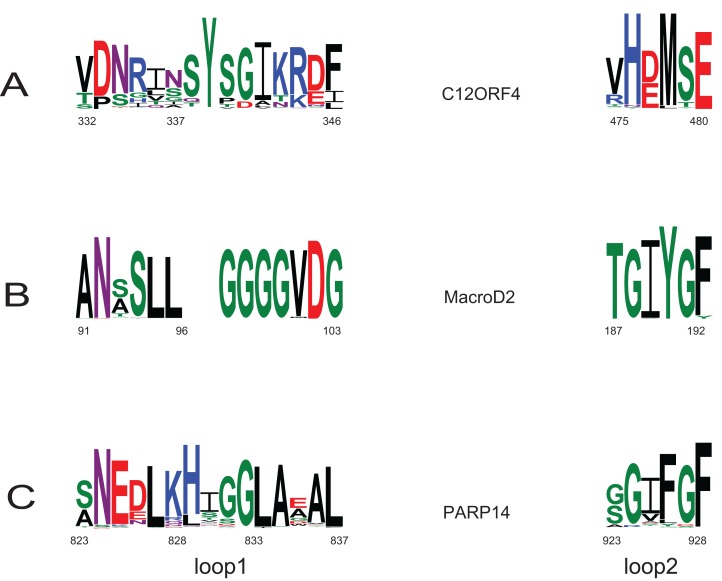
Sequence logos for active sites in loop 1 and loop 2 in two Macro families: MacroD2 and PARP14 compared to DUF2362. Conservation of catalytic residues in multiple sequence alignment constructed for MacroD2 and PARP14 homologs and corresponding conserved residues in aligned fragments of DUF2362. Amino acid position numbering according to human representatives for all three sequence groups.

Investigation of likely domain structure in uncharacterized proteins can be aided by the analysis of their amino acid sequence logo, a graphical representation of conservation of sequence positions in the protein family. A logo created using multiple alignment of the DUF2362 family shows strong sequence conservation in the C-terminal region, but also some strongly conserved motifs between positions 85 and 110 and in the region 230–260 (see Fig. 4 and Fig. S1 for full-length DUF2362 logo). Two Asp residues (D333 and D345) identified in profile–profile alignments (Fig. 1) as likely counterparts of MacroD2 catalytic residues are well-conserved in the family, although consensus does not reach 100% (Fig. 5). The long region following the putative catalytic motifs is the least conserved part of the DUF2362 alignment (Fig. 3). This region–378–408 in human C12ORF4–has no counterparts in known Macro domain structures, it is also very poorly conserved in the DUF2362 family (Fig. S1). Figure 5 presents a comparison of conservation within two catalytic loops in two Macro domain families–eraser-type (MacroD2) and reader-type (PARP14)–and in the corresponding regions of DUF2362. In C12ORF4, two conserved Asp residues, D333 and D345, are putative counterparts of MacroD2 active site N92 and D102. While similarities within loop 1 in C12ORF4 and two known Macro families are quite noticeable, the loop 2 motifs are more different. The presence of highly conserved methionine and histidine is noteworthy. These residues may be involved in binding/positioning of the distal ribose of ADPr, similarly to Y190 of MacroD2 and F926 of PARP14.

Apart from the predicted ADPr binding and catalytic site, the functions of conserved residues are less obvious. Several strikingly well-conserved cysteine residues could serve the building of disulfide bridges. The very well-conserved histidine 431 corresponds to a histidine conserved in a VIH[TA] motif in Macro domains of PARP14 and MacroD2 and may be involved in the positioning of the active site Asp. The most conserved region (475–520) of the DUF2362/C12ORF4 family corresponds to helices 4 and 5 in known Macro domains which are distal to the ADP-ribose site and whose functions are not clear.

**Figure 6:**
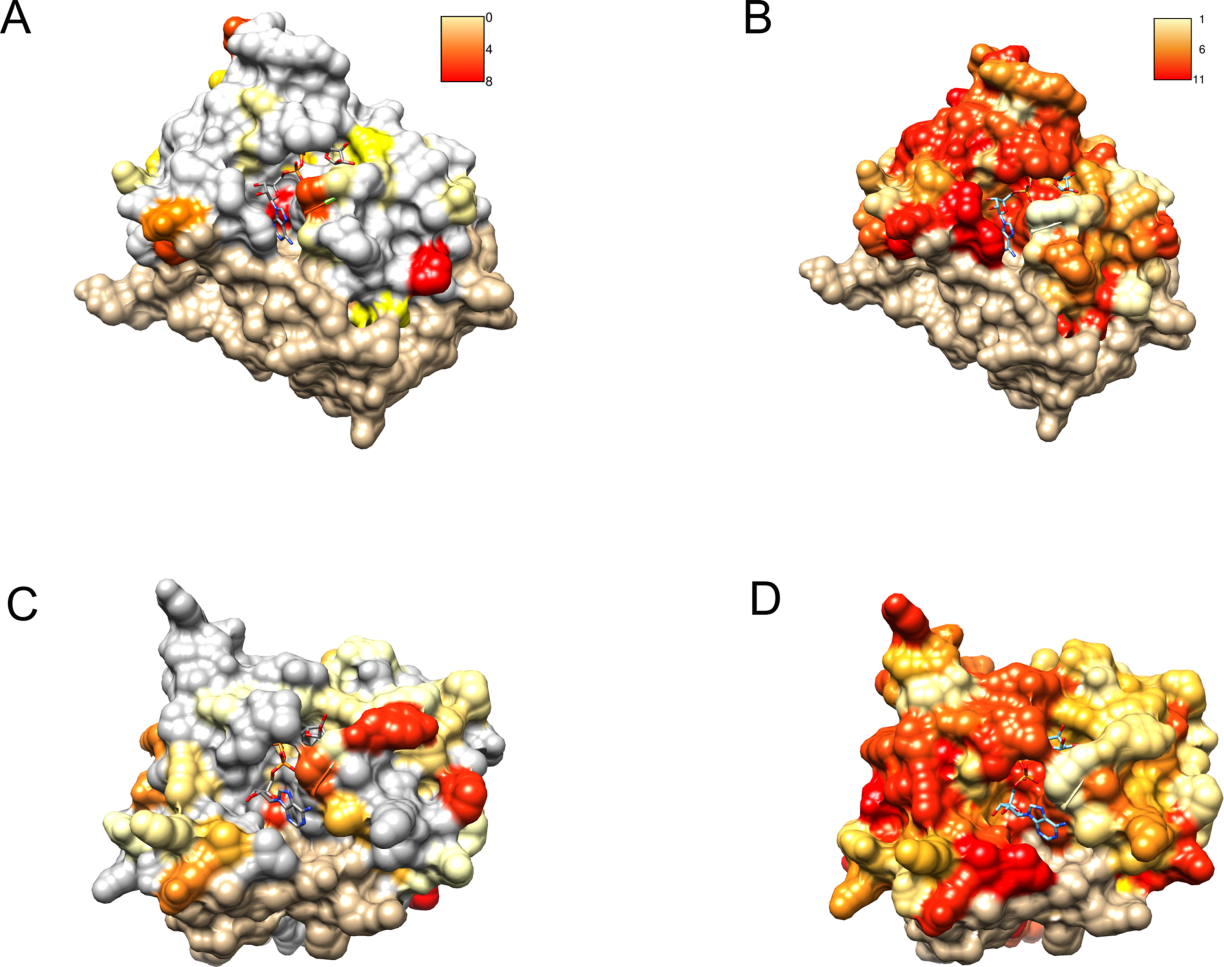
Similarity of DUF2362 to known Macros and sequence variation within the family mapped on three-dimensional structures. Bound ADPr molecules shown. (A) Conservation in human MacroD2/C12ORF4 pairwise sequence alignment mapped onto MacroD2 structure (PDB 4IQY). Coloring of the molecular surface according to BLOSUM62 values for alignment positions (red–high similarity, yellow–low similarity, white–no similarity (BLOSUM62 values below zero), beige–unaligned regions). (B) Conservation in the DUF2362 family sequence alignment mapped onto MacroD2 structure (PDB 4IQY). Coloring of the molecular surface: Jalview-derived alignment conservation value (red–high conservation, yellow–low conservation, white–no similarity, beige–unaligned regions. (C) Conservation in human PARP14/C12ORF4 pairwise sequence alignment mapped onto PARP14 structure (PDB 3Q6Z). Coloring as in (A). (D) Conservation in the DUF2362 family sequence alignment mapped onto PARP14 structure (PDB 3Q6Z). Coloring as in (B).

Profile–profile alignment methods offer automatic pipelines enabling modeling of the putative three-dimensional structure of the query protein. In the case of C12ORF4, automated methods would be quite risky, mainly due to the presence of long insertions (likely loops) within the hypothetical Macro domain. Residues from 374 to 408 had to be modeled without a structural template. Secondary structure prediction run for amino acids in this region of C12ORF4 indicate a mainly unstructured character of this fragment, so we decided to build a preliminary model for the C12ORF4 structure in the form of a homology model based on a human MacroD2 template, using our own manually adjusted alignment, with the building and optimization of missing loops using the I-TASSER server. An “eraser” template (PDB: 4IQY) was chosen because of the fact that only one Macro-like domain was detected in our query. This is similar to the “eraser” MacroD1/2 proteins that have only one catalytically active Macro domain. In contrast, the “reader” PARP14 contains three repeated Macro domains. Also, there are two likely catalytic Asp residues present in the predicted loop 1 of C12ORF4. Before building the structure model, we analyzed possible templates and conservation of important residues in C12ORF4 and the whole DUF2362 family, using sequence alignments and logo. As can be noticed in Fig. 6, conservation of DUF2362 alignment positions matched to the ligand binding cleft of MacroD2 and PARP14 is high (Figs. 6B and 6D). Also, considering pairwise alignment mapping (Figs. 6A and 6C), one can see that apart from conserved (Asp/Asp) or almost conserved (Asn/Asp) catalytic positions there are also some residues conserved in C12ORF4 in the neighborhood of the putative ADPr binding site–proline, alanine and glycine in the case of both templates and tyrosine in the case of PARP14.

**Figure 7:**
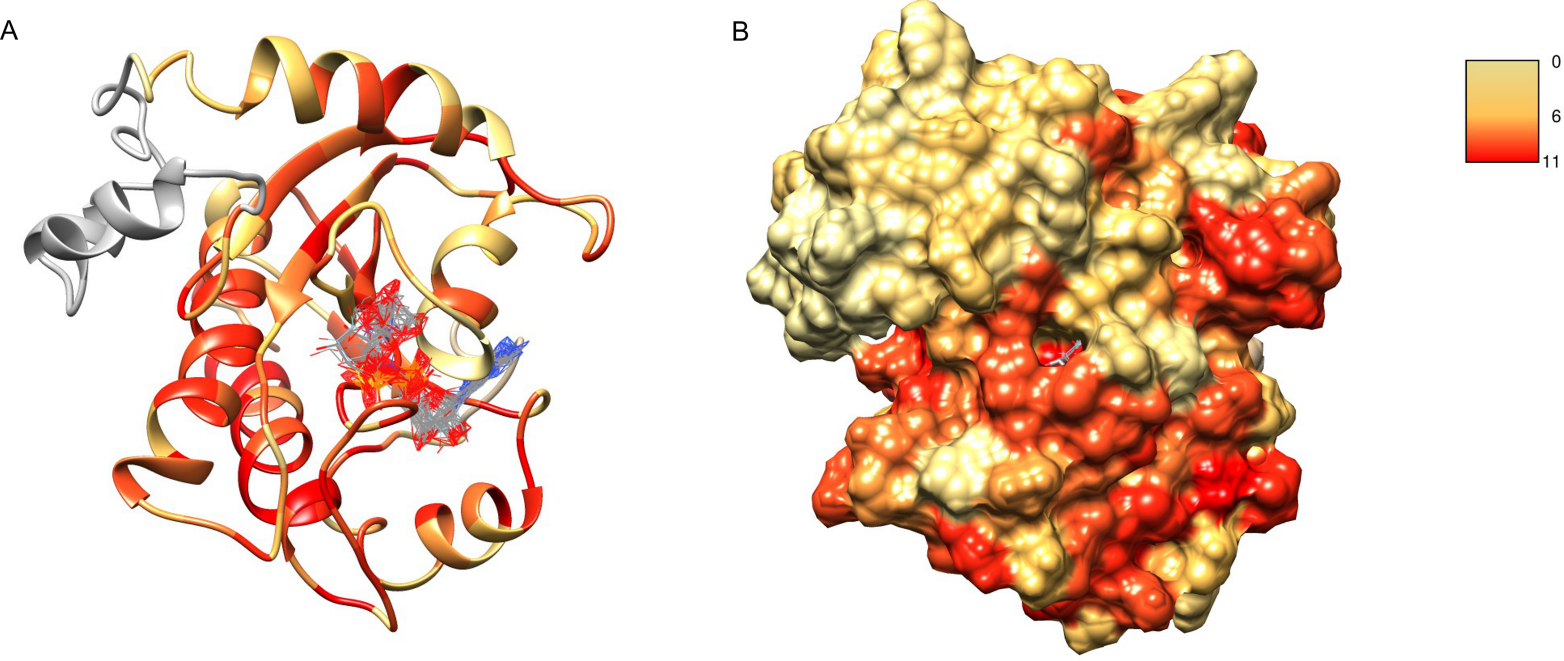
Structure model of C12ORF4 with COFACTOR positions of high scoring potential ligands–ADP-ribose molecules. Structure coloring: Jalview-derived sequence alignment conservation value for the DUF2362 family (red–high conservation, yellow–low conservation. (A) Ribbon representation (the region 378–408 modeled by I-Tasser server without template depicted in gray). (B) Molecular surface map.

**Figure 8:**
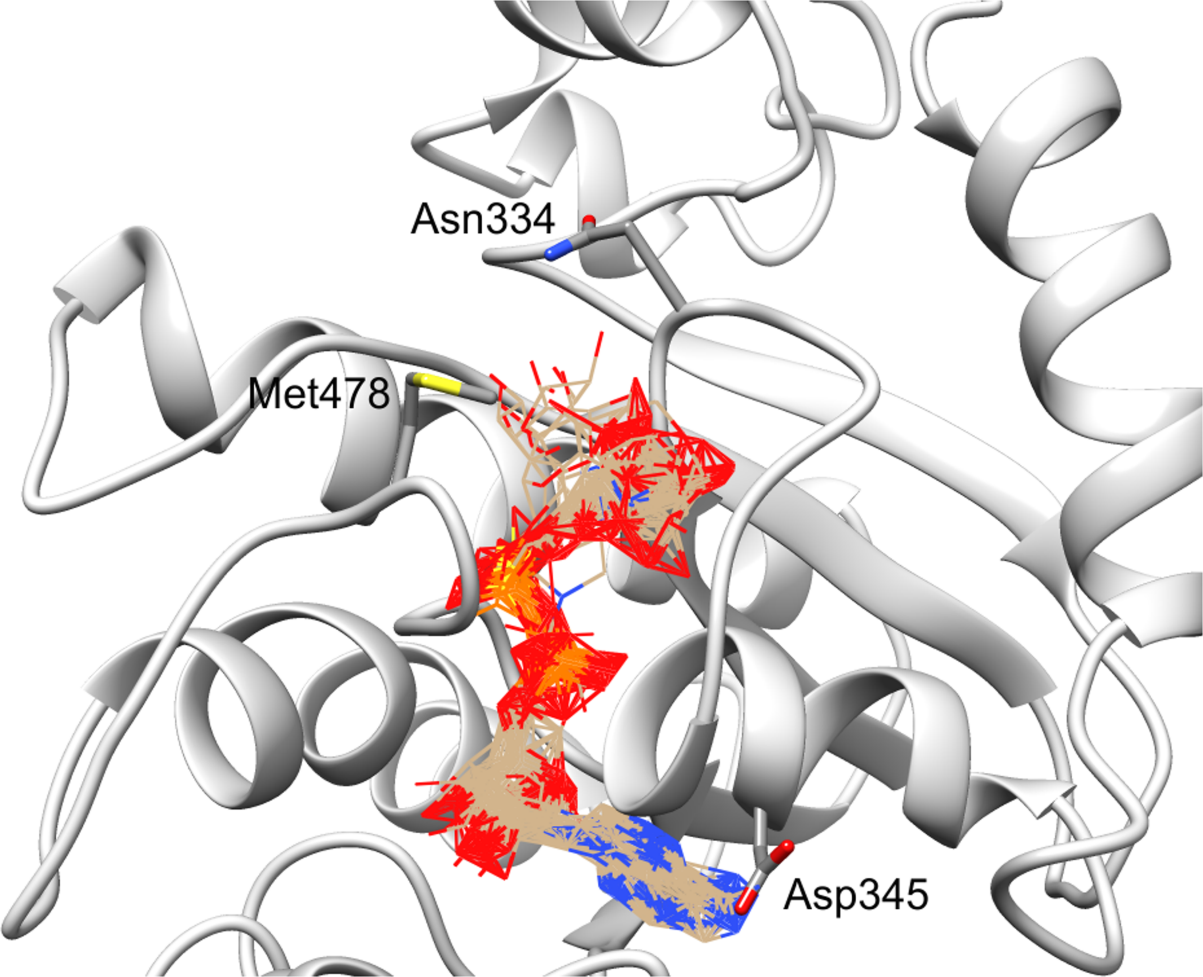
Ligand binding site in the structure model of C12ORF4 with hypothetical positions of predicted ligands–ADP-ribose molecules. Ribbon representation with Asn 334, Asp 345 in loop 1 and Met 478 in loop 2 (identified as counterparts of MacroD2 catalytic residues) depicted in the all-atom representation.

After obtaining the structure model of human C12ORF4, with the ab initio constructed fragment, we used the COFACTOR algorithm to identify and place putative ligands within the obtained model. COFACTOR scans the PDB database for structures similar to the query and containing co-crystalized ligands to propose possible ligand binding site locations within the analyzed model. This represents an approximate result and one should not treat the obtained ligand binding poses as certain, but one can rank proposed ligands and identify clefts likely responsible for its binding. Results shown in Table S1 support ADPr binding by C12ORF4. The highest scoring COFACTOR ligands are ADP-riboses, which are frequently co-crystalized with Macro domain structures, which is not surprising considering the template used for homology modeling. The predicted ADPr binding cleft is localized between loop 3 (counterpart of MacroD loop 2) and helix 5 and flanked by the loop modeled by I-TASSER (see Figs. 7 and 8). Hydrogen bonds to ADPr detected in the model using UCSF Chimera tools (Pettersen et al., 2004) involve Ile 524, and His, Asp and Met of HDM (476–478) motif aligned to MacroD2 catalytic tyrosine. However, a detailed investigation of ligand binding based on the preliminary model and COFACTOR results would be too speculative.

Structure modeling for uncharacterized proteins is always a risky enterprise when no highly homologous template is available. In the case of C12ORF4, an additional complication is the presence of a putative N-terminal domain and a long insertion within the Macro-like region.

### Biological function of C12ORF4

The only report available that investigated the molecular function of C12ORF4 is that of Mazuc et al. (2014) who used an intrabody approach to identify proteins involved in FcεRI-induced mast cell degranulation. They showed that silencing C12ORF4 influenced FcεRI-mediated signaling events; namely, it caused a decrease in FcεRI-mediated tyrosine phosphorylation of Src and Syk tyrosine kinases as well as the downstream kinases, MAPKs and Akt. This, in turn, had a clear effect on degranulation, by decreasing the release of TNF-α and β-hexaminidase.

The Mazuc study, together with our findings that link C12ORF4 to ADP-ribosylation, casts interesting light on the known function of PARP-1 in degranulation, and specifically in modulating asthma-associated production of cytokines (Sethi, Dharwal & Naura, 2017). Studies on asthma patient material as well as animal models have led to inhibitors of PARP1 and PARP14 being considered for asthma treatment and being investigated in animal models (Zaffini, Gotte & Menegazzi, 2018). For example, in an early study in guinea pig, PARP inhibition prevented an induced asthma-like reaction (Suzuki et al., 2004). Allergen exposition induced a rapid increase in PARP activity, bronchiolar constriction, pulmonary air space inflation and mast cell degranulation in sensitized animals, while inhibition of PARP prevented the above morphological and biochemical changes to lung tissue (Suzuki et al., 2004). Our results allow us to propose a hypothesis that Macro-like C12ORF4, as a putative ADPr “eraser,” regulates PARP-mediated ADP-ribosylation signaling, similarly to PARG, whose role in countering PARP action in airway disease is known (Sethi, Dharwal & Naura, 2017). The fact that C12ORF4 expression has been found to affect phosphorylation of several kinases (Mazuc et al., 2014) allows speculation that the effects of PARP and C12ORF4 on degranulation may be mediated by direct ADP-ribosylation of kinases. There are known cases of regulation of kinase signaling pathways by direct ADP-ribosylation of key kinases, by effectors from infectious bacteria (Young et al., 2014) and also by endogenous human ADP-ribosyltransferases (Song et al., 2016; Li et al., 2018).

An interesting connection between the degranulation-related phenotype attributed to C12ORF4 and its relationship to ID is the fact that mast cell degranulation is one of the processes involved in brain inflammation (Dong et al., 2017; Skaper et al., 2018). Brain inflammation, in turn, can lead to ID and related disorders (Di Marco et al., 2016).

Exploitation of in silico databases for additional clues regarding C12ORF4 functions provides rather few hints. According to databases such as Expression Atlas (Papatheodorou et al., 2018), Human Protein Atlas (Uhlen et al., 2015) and Human Proteome Map (Kim et al., 2014), the C12ORF4 protein-coding gene (on a protein and transcript level) is expressed in most normal tissues, with a high level of expression in the brain and nerves, and in the genitals. In disease-specific studies, high expression levels of C12ORF4 have been reported for samples collected from glioblastoma multiforme and glioma, lymphoma, pancreatic adenocarcinoma, and ovarian adenocarcinoma (Cancer Genome Atlas Research Network et al., 2013). The cBioPortal database (Cerami et al., 2012) gives evidence that the C12ORF4 protein-coding gene is altered (mutation, but not deletion) in 3% of 1,939 cases (59 patients) of endometrial cancer, and smaller fractions of patients with other malignancies, for example, breast cancer, bladder cancer, colorectal cancer and melanoma. The more frequently occurring alterations of the C12ORF4 protein-coding gene are presented in Table S2. The most frequent mutation, missense R335Q, has been observed in 14 cases, mainly in uterine endometrioid carcinoma samples, but also in patients with other malignancies (cBioportal). Arg335 is located within the putative Macro domain region of C12ORF4, in the conserved motif DNR, containing Asp333, suggested by us to be a likely catalytic residue (Fig. 5).

According to tools for elucidation of biological relationship networks, such as Ingenuity Pathway Analysis (https://www.qiagenbioinformatics.com/products/ingenuity-pathway-analysis) and String (Szklarczyk et al., 2015), C12ORF4 is involved in a functional network involving a number of mostly cytoplasmic signaling proteins. One of these interactors, TBCK, described as a modulator of mammalian TOR signaling, is involved in the regulation of cell proliferation and growth and control of actin-cytoskeleton organization. Interestingly, mutations in TBCK lead to a syndrome of ID (Bhoj et al., 2016). Other interactors include the interleukin receptor IL6R, that is a proposed asthma risk locus (Ferreira et al., 2011) and is functionally related to a number of degranulation-related signaling mediators (see Fig. S2)

## Conclusions

In summary, we present strong in silico evidence that DUF2362 belongs to the Macro clan/superfamily. The similarity to known Macro domains is clear and suggestive. It remains unclear whether this new Macro domain has a catalytic activity or only a “reader” function. However, several lines of evidence support the catalytic, not pseudoenzyme function, including conservation of a putative active site.

Turning a structure prediction into a useful molecular function and biological process prediction is always a challenge. Here, we approached the DUF2362/C12ORF4 family from several angles, trying to combine structural prediction with available functional data. The final functional answers, nevertheless, will only come from experiments, both biochemical and biological, that should cast full light on C12ORF4, an intriguing putative novel addition to eukaryotic ADP-ribosylation signaling. Thus, until decisive biochemical and structural biology data provide unequivocal functional validation, DUF2362 has to be regarded as a tentative new member of the Macro clan/superfamily.

## Supplemental Information

10.7717/peerj.6863/supp-1 Supplemental Information 1 Figure S1. Full length sequence logo for the DUF2362 family . The macro domain region 322–545 of this logo is used for Fig. 3, logo positions are numbered according to human sequence of C12ORF4.

10.7717/peerj.6863/supp-2 Supplemental Information 2 Figure S2. Biological relationship network of human C12ORF4. Biological relationships of human C12ORF4, including direct relationships (shown as blue lines) and secondary interactions, as derived from the Ingenuity database. Network is augmented with proteins providing shortest network paths to Degranulation of mast cells (shown as blue lines).

10.7717/peerj.6863/supp-3 Supplemental Information 3 Table S1. COFACTOR results for C12ORF4 structure model. Top three hits of COFACTOR - structure based function predictions algorithm of I-Tasser server for putative C12ORF4 structure model. Cscore is the confidence score of predicted GO terms. Cscore is the confidence score of predicted binding site with values range in between [0 and 1]; where a higher score indicates a more reliable ligand-binding site prediction.

10.7717/peerj.6863/supp-4 Supplemental Information 4 Table S2. Alterations of gene C12ORF4 observed in different types of cancer tissues. According to cBioPortal data, PanCancer studies (Cerami et al., 2012; Gao et al., 2013).

10.7717/peerj.6863/supp-5 Supplemental Information 5 Data S1. Multiple sequence alignment for DUF2362 family. The alignment is based on sequences selected from Pfam database for DUF2362 representatives, column numbering according to human sequence of C12ORF4. Source file for DUF2362 sequence logos (Figs. 3 and 4; Fig. S1).

10.7717/peerj.6863/supp-6 Supplemental Information 6 Data S2. CLANS clustering analysis file. CLANS clustering analysis file, corresponding to Fig. 4.

10.7717/peerj.6863/supp-7 Supplemental Information 7 Data S3. Structure model of human C12ORF4, PDB format. Structure model of human C12ORF4, PDB file, used for Figs. 6 and 7.

## Additional Information and Declarations

### Data Availability

The following information was supplied regarding data availability: The raw data (full results of bioinformatics analyses) is available as Supplementary Files.
