# Supplementary figures and images for: A novel conserved family of Macro-like domains—putative new players in ADP-ribosylation signaling
Source: PeerJ. 2019 May 1;7:e6863. doi: 10.7717/peerj.6863 (PMC6500376; doi:10.7717/peerj.6863)

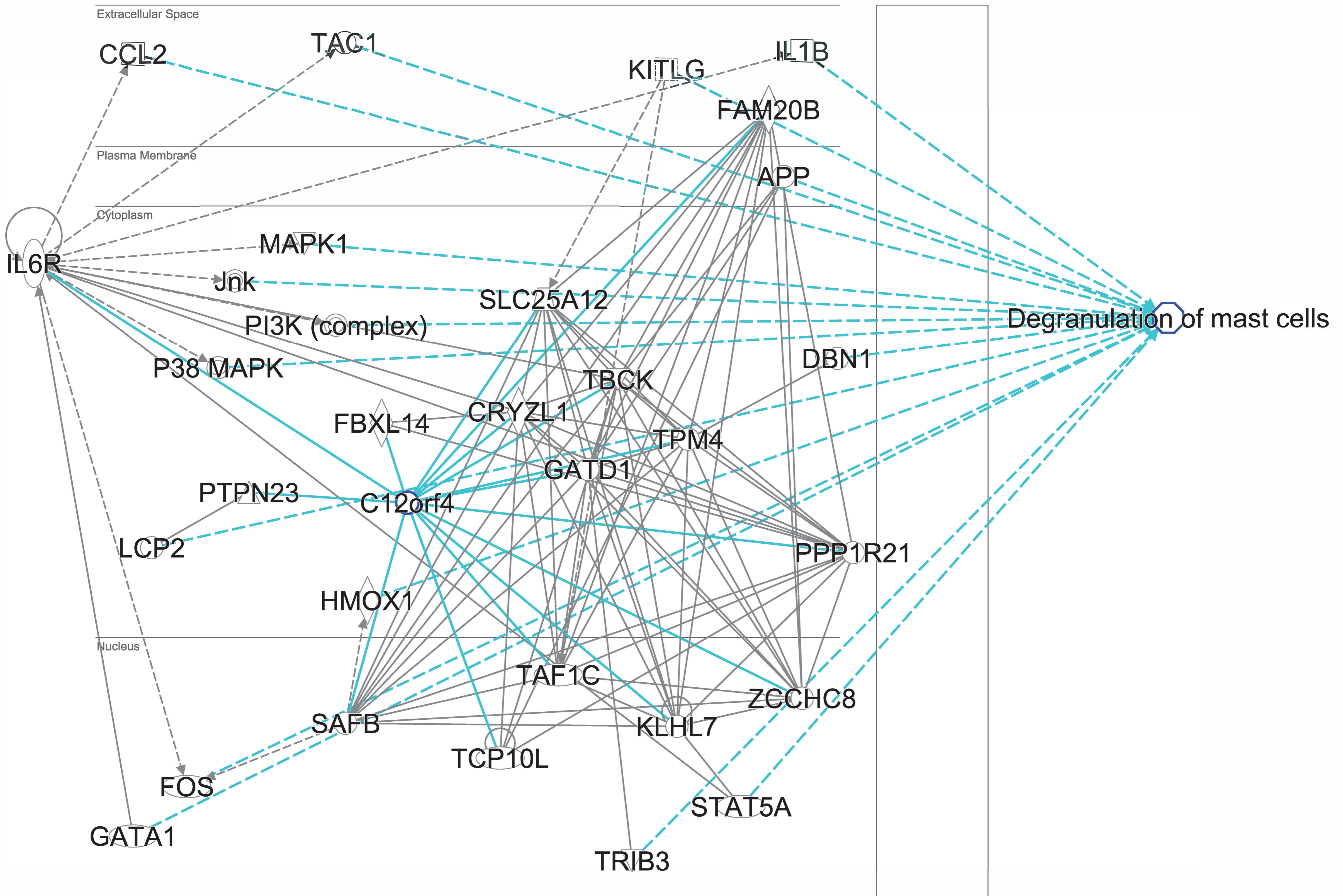

Supplement: Supplemental Information 2 — Biological relationships of human C12ORF4, including direct relationships (shown as blue lines) and secondary interactions, as derived from the Ingenuity database. Network is augmented with proteins providing shortest network paths to Degranulation of mast cells (shown as blue lines). [file peerj-07-6863-s002.pdf]
