# Supplementary material for: A novel conserved family of Macro-like domains—putative new players in ADP-ribosylation signaling
Source: PeerJ. 2019 May 1;7:e6863. doi: 10.7717/peerj.6863 (PMC6500376; doi:10.7717/peerj.6863)
Supplement: Supplemental Information 3 — Top three hits of COFACTOR - structure based function predictions algorithm of I-Tasser server for putative C12ORF4 structure model. Cscore is the confidence score of predicted GO terms. Cscore is the confidence score of predicted binding site with values range in between [0 and 1]; where a higher score indicates a more reliable ligand-binding site prediction. [file peerj-07-6863-s003.docx]

| **Rank** | **C-score** | **Cluster size** | **PDB Hit** | **Ligand name** | **Ligand Binding Site Residues** |
| --- | --- | --- | --- | --- | --- |
|  |  |  |  |  |  |
| 1 | 0.36 | 35 | 5cms | APR (ADP-RIBOSE) | 6,7,8,16,17,109,111,147,148,149,150,151,152,153,196 |
| 2 | 0.03 | 3 | 4tu0 | Nucleic acid | 16,17,147,150,151,152,193,207,210 |
| 3 | 0.04 | 4 | 4ej7 | CA | 97,110 |
