# Supplementary material for: A novel conserved family of Macro-like domains—putative new players in ADP-ribosylation signaling
Source: PeerJ. 2019 May 1;7:e6863. doi: 10.7717/peerj.6863 (PMC6500376; doi:10.7717/peerj.6863)
Supplement: Supplemental Information 4 — According to cBioPortal data, PanCancer studies (Cerami et al., 2012; Gao et al., 2013). [file peerj-07-6863-s004.docx]

|  | Brest cancer | | Non-small Cell Lung Cancer | | Ovarian Cancer | | Endometrial Cancer | | Esophagogastric cancer | | Bladder Cancer | |
| --- | --- | --- | --- | --- | --- | --- | --- | --- | --- | --- | --- | --- |
|  | % | cases | % | cases | % | cases | % | cases | % | cases | % | cases |
| mutation | 0,2 | 12 | 0,32 | 22 |  |  | 3,04 | 59 | 0,65 | 21 | 0,69 | 13 |
| fusion | 0,02 | 1 |  |  | 0,05 | 1 |  |  |  |  |  |  |
| amplification | 1,78 | 106 | 1,1 | 77 | 5,82 | 116 | 1,08 | 21 | 0,65 | 21 | 0,9 | 17 |
| deep deletion | 0,13 | 8 | 0,33 | 23 | 0,05 | 1 | 0,15 | 3 | 0,34 | 11 | 0,26 | 5 |
|  | B-Lymphoblastic Leukemia/Lymphoma | | Glioma | | Melanoma | | Colorectal cancer | | Pancreatic Cancer | | Prostate Cancer | |
|  | % | cases | % | cases | % | cases | % | cases | % | cases | % | cases |
| mutation |  |  | 0,11 | 4 | 1,05 | 18 | 0,47 | 19 | 0,12 | 2 | 0,02 | 1 |
| fusion |  |  |  |  |  |  |  |  |  |  |  |  |
| amplification | 0,46 | 9 | 0,66 | 25 | 0,29 | 5 | 0,1 | 4 | 1,05 | 18 | 0,16 | 8 |
| deep deletion | 1,16 | 23 | 0,03 | 1 | 0,12 | 2 |  |  | 0,06 | 1 | 0,24 | 12 |
