# Supplementary material for: A novel conserved family of Macro-like domains—putative new players in ADP-ribosylation signaling
Source: PeerJ. 2019 May 1;7:e6863. doi: 10.7717/peerj.6863 (PMC6500376; doi:10.7717/peerj.6863)
Supplement: Supplemental Information 1 — The macro domain region 322–545 of this logo is used for Fig. 3, logo positions are numbered according to human sequence of C12ORF4. [file peerj-07-6863-s001.pdf]

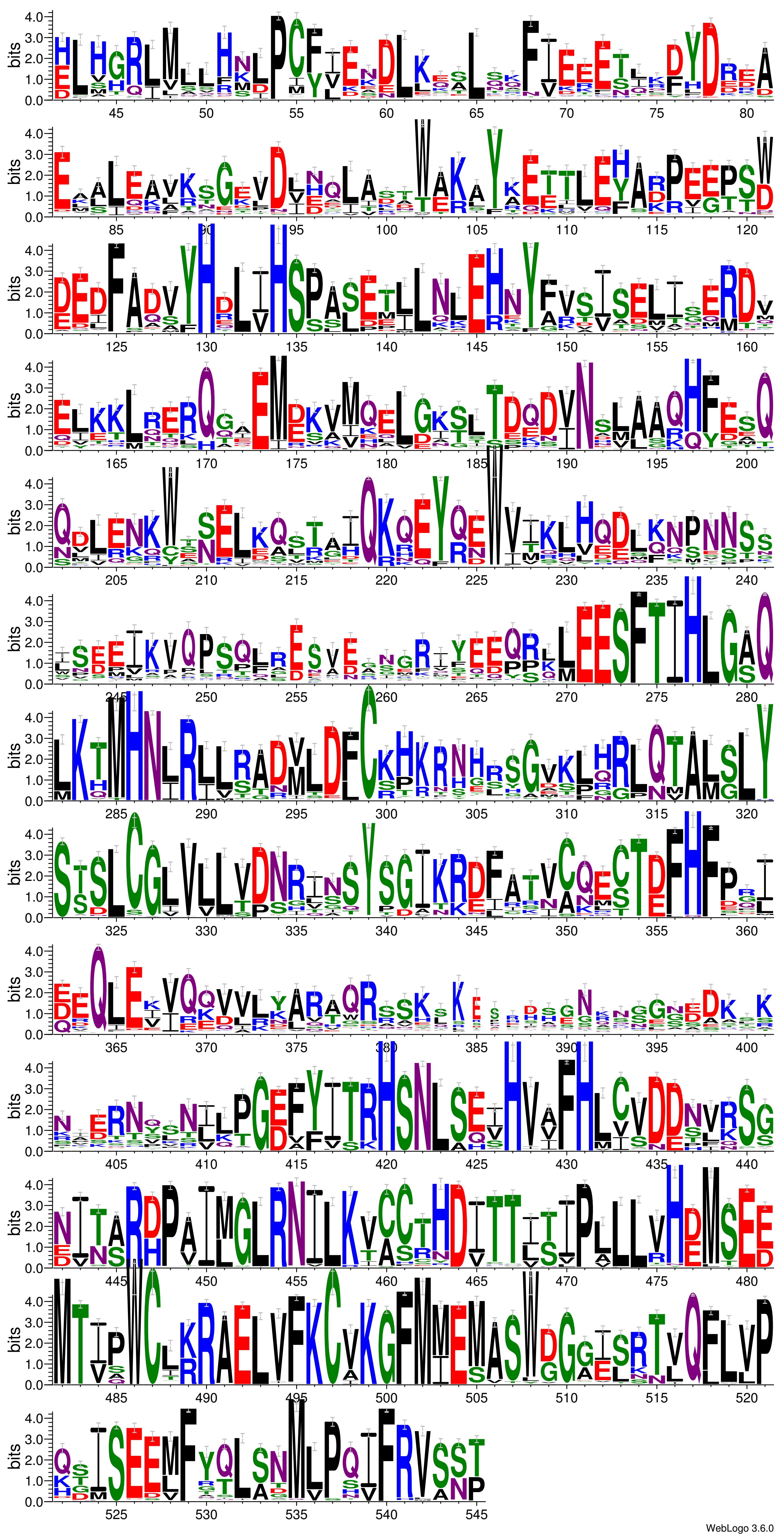
bits
WebLogo 3.6.0
